# Influences of phase transition and microstructure on dielectric properties of Bi_0.5_Na_0.5_Zr_1-x_Ti_x_O_3 _ceramics

**DOI:** 10.1186/1556-276X-7-45

**Published:** 2012-01-05

**Authors:** Panupong Jaiban, Ampika Rachakom, Sukanda Jiansirisomboon, Anucha Watcharapasorn

**Affiliations:** 1 Department of Physics and Materials Science, Faculty of Science, Chiang Mai University, Chiang Mai 50200, Thailand; 2 Materials Science Research Center, Faculty of Science, Chiang Mai University, Chiang Mai 50200, Thailand

**Keywords:** ceramics, X-ray diffraction, electron microscopy, crystal structure, electrical properties

## Abstract

Bismuth sodium zirconate titanate ceramics with the formula Bi_0.5_Na_0.5_Zr_1-x_Ti_x_O_3 _[BNZT], where *x *= 0.3, 0.4, 0.5, and 0.6, were prepared by a conventional solid-state sintering method. Phase identification was investigated using an X-ray diffraction technique. All compositions exhibited complete solubility of Ti^4+ ^at the Zr^4+ ^site. Both a decrease of unit cell size and phase transition from an orthorhombic Zr-rich composition to a rhombohedral crystal structure in a Ti-rich composition were observed as a result of Ti^4+ ^substitution. These changes caused dielectric properties of BNZT ceramics to enhance. Microstructural observation carried out employing SEM showed that average grain size decreased when addition of Ti increased. Grain size difference of BNZT above 0.4 mole fraction of Ti^4+ ^displayed a significant increase of dielectric constant at room temperature.

## Background

Nowadays, materials possessing a diffuse phase transition at high temperature are of interest because they are believed to be a promising candidate for various electronic devices. Examples are multilayer capacitors, detectors, MEMs, sensors, actuators, etc. However, high permittivity at room temperature is also significant. Recently, Lily et al. [1] have successfully fabricated and investigated a novel perovskite-type ceramic of Bi_0.5_Na_0.5_ZrO_3 _[BNZ] compound. They reported that the mentioned ceramic had an orthorhombic structure and a high curie temperature of 425°C. This value is rather high when compared with well-known lead-free ceramics such as BaTiO_3 _(130°C) [2] and Bi_0.5_Na_0.5_TiO_3 _[BNT] (320°C) [3]. Unfortunately, the BNZ system showed low dielectric constant at room temperature, i.e., approximately 100, 60, and 25 at a frequency of 1, 10, and 100 kHz, respectively.

According to the most investigated PbTiO_3_-PbZrO_3 _[PZT] solid solution system, it was known that the dielectric constant of orthorhombic PbZrO_3 _compound was quite low (i.e., approximately 190) [4], but the value could be enhanced to range about 400 to 800 with partial substitution of Ti^4+ ^ions at the Zr^4+ ^site within the perovskite lattice [5]. Improvement of the permittivity was attributed to the transformation of orthorhombic crystal structure to rhombohedral and tetragonal lattices. In this phase transformation, the Zr/Ti ratio was the main factor that specified the crystal structure of PZT ceramics.

For a similar system of BNT-BNZ, Yamada et al. [6] predicted only that the phase-transition point of the phase diagram seemed to be approximately at a Zr/Ti ratio of 0.6:0.4. In addition, a study concerning Bi_0.5_Na_0.5_Zr_1-x_Ti_x_O_3 _[BNZT] ceramic from a Zr-rich composition has not been reported. Hence, the purpose of this work is to investigate influences of the occupancy of Ti^4+ ^ions at the B-site of Zr^4+ ^host ions with Zr/Ti ratios of 0.7:0.3, 0.6:0.4, 0.5:0.5, and 0.4:0.6 on phase transition and dielectric properties at room temperature of the orthorhombic BNZ ceramic.

## Methods

The specimen was fabricated according to the chemical formula Bi_0.5_Na_0.5_Zr_1-x_Ti_x_O_3_, where *x *= 0.3, 0.4, 0.5, and 0.6. The powders were prepared by a conventional mixed-oxide method. The starting materials used in this study were ZrO_2 _(99%, Riedel-de Haën, Sigma-Aldrich Corporation, St. Louis, MO, USA), TiO_2 _(99%, Riedel-de Haën), Bi_2_O_3 _(98%, Fluka, Sigma-Aldrich Corporation, St. Louis, MO, USA), and Na_2_CO_3 _(99.5%, Riedel-de Haën). The mixtures of oxides were ball milled in ethanol for 24 h. The mixed powders were dried at 120°C for 24 h and then calcined in a closed alumina crucible at a temperature of 800°C for 2 h with a heating/cooling rate of 5°C/min. After sieving, a few drops of 3 wt.% polyvinyl alcohol binders were added to the mixed powders which were subsequently pressed into pellets with a diameter of 10 mm using a uniaxial press with 1-ton weight for 15 s. Binder removal was carried out by heating the pellets at 500°C for 1 h. These pellets were then sintered at 950°C for a 2-h dwell time with a heating/cooling rate of 5°C/min on a covered alumina plate.

The sintered samples were polished using sandpaper and cleaned using an ultrasonic bath. After that, phase identification of ceramics was investigated in a 2*θ *range of 20° to 80° using an X-ray diffractometer [XRD] (Phillip Model X-pert, PANalytical B.V., Almelo, The Netherlands). For a microstructural observation, the sintered pellets were polished using sandpaper as well as alumina slurry and cleaned in the same ultrasonic bath. Then, the polished samples were etched at a temperature of 800°C for 15 min with a heating/cooling rate of 5°C/min on a covered alumina plate. Microstructure of etched materials was observed using a backscattered-electron mode of a scanning electron microscope [SEM] (JSM 6335F, JEOL Ltd., Akishima, Tokyo, Japan).

Numerical detail of the lattice parameters of all samples was obtained from fitting between observed reflection angles of experimental XRD patterns and calculated angles using the Powder Cell Software (BAM, Berlin, Germany) [7]. Measurement of grain size was performed by employing a linear intercept method on SEM images. For dielectric property measurements, the sintered samples were polished by sandpaper until the thickness was approximately 1 μm. Subsequently, two parallel surfaces of polished ceramics were painted with a silver paste for electrical contacts. Dielectric constant and loss were measured at room temperature with measured frequencies of 1, 10, and 100 kHz using a 4284A LCR meter (Agilent Technologies Inc., Santa Clara, CA, USA).

## Results and discussion

**Figure 1:**
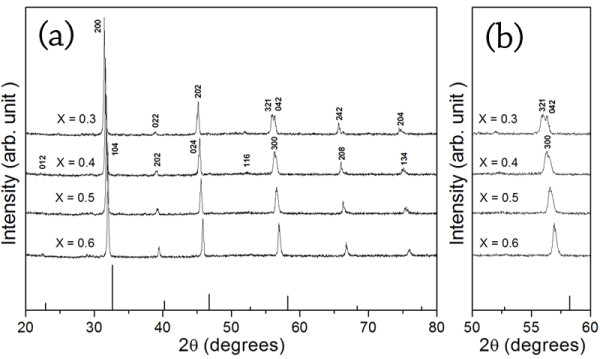
**X-ray diffraction patterns of BNZT ceramics**. The samples were sintered at 950°C; (**a**) 2*θ *= 20° to 80° and (**b**) 2*θ *= 50° to 60°.

**Figure 2:**
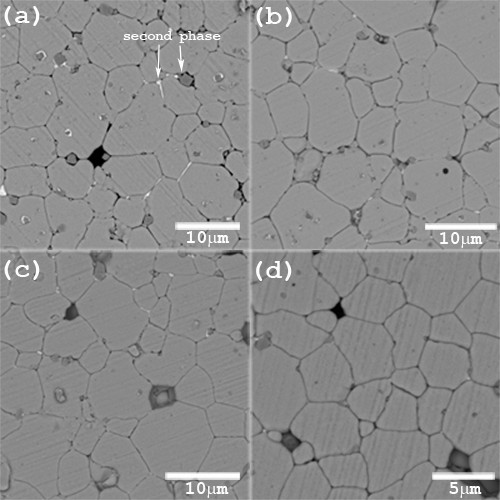
**SEM micrographs of BNZT ceramics**. The samples were sintered at 950°C; (**a**) *x *= 0.3, (**b**) *x *= 0.4, (**c**) *x *= 0.5, and (**d**) *x *= 0.6.

Figure 1 presents XRD patterns of Bi_0.5_Na_0.5_Zr_1-x_Ti_x_O_3 _ceramics where *x *= 0.3, 0.4, 0.5, and 0.6. All compositions exhibited a perovskite structure and complete solubility. As observed, peaks in XRD patterns shifted to higher reflection angles when Ti addition increased. The analysis indicated that Ti^4+ ^could diffuse successfully into the BNZ lattice to form desired solid solutions. Smaller ion of Ti^4+ ^(0.605 Å) substituting a larger host ion of Zr^4+ ^(0.72 Å) [8] at the B-site of the BNZ perovskite material resulted in a decrease in volume of its original unit cell. This therefore caused the patterns to shift to the right. Besides, modification by adding more than 0.4 mole fraction of Ti^4+ ^changed the crystal system from an orthorhombic prototype structure to another structure. The feature of the changed patterns was in agreement with the rhombohedral structure of BNT at room temperature (ICSD file no. 28-0983). The presence of the rhombohedral structure was believed to be a Ti-rich composition in the BNZ-BNT phase diagram. Observed planes in the 2*θ *range of 50° to 60° include (321), (042), and (300) as shown in Figure 2b. For a Ti^4+ ^amount of 0.3 mole fraction, the BNZT ceramic maintained the orthorhombic structure with splitted peaks, i.e., (321) and (042). Subsequently, the existence of a single peak (300) was found for the composition where *x *= 0.4. The orthorhombic to rhombohedral phase transition was then presumed to occur at a Bi_0.5_Na_0.5_Zr_0.6_Ti_0.4_O_3 _composition at room temperature. This was influenced by the distortion of the crystal lattice because Ti^4+ ^occupied at the Zr^4+ ^site. The phase transition for the Zr/Ti ratio (0.6:0.4) found in this study was in agreement with the previous report of Yamada et al. [6] who mentioned that the approximate phase transition point of the BNT-BNZ binary system was at a Zr/Ti ratio of 0.6:0.4. Quantitative data of lattice parameters obtained from the comparison between the observed and calculated reflection angles with a selected d-spacing are also given in Table 1. Thus, as a result, an isovalent substitution of Ti ion not only reduced the unit cell dimension, but also promoted the phase transition at the composition of Bi_0.5_Na_0.5_Zr_0.6_Ti_0.4_O_3_.

**Table 1:** Lattice parameters and grain size of BNZT ceramics

*x*	(hkl)	2*θ*_obs_	2*θ*_cal_	Lattice parameters	Grain size (μm)
0.3	(042)	56.13	56.10	*a *= 5.6893 Å *b *= 8.0434 Å *c *= 5.6553 Å *α *= 90°	5.65 ± 1.63
0.4	(300)	56.51	56.52	*a *= 3.9875 Å; *α *= 89.9247°	5.55 ± 1.84
0.5	(300)	56.61	56.60	*a *= 3.9835 Å; *α *= 89.8975°	5.07 ± 1.57
0.6	(300)	56.97	56.97	*a *= 3.9602 Å; *α *= 89.8713°	3.76 ± 1.24

*x*, amount of Ti^4+^; 2*θ*_obs_, observed reflection angle; 2*θ*_cal_, calculated reflection angle.

SEM-BEI images of Bi_0.5_Na_0.5_Zr_1-x_Ti_x_O_3 _ceramics, where *x *= 0.3, 0.4, 0.5, and 0.6, are shown in Figure 2. All compositions produced similarly shaped crystalline grains. The images also showed that the average size of grains decreased slightly with an increase of the Ti content up to 0.5 mole fraction and decreased sharply for the Bi_0.5_Na_0.5_Zr_0.4_Ti_0.6_O_3 _specimen. The mentioned analysis suggested that Ti addition also affected the microstructure of BNZT materials. Furthermore, in Figure 2a, a weak trace of secondary phases was observed for the sintered specimen with the Bi_0.5_Na_0.5_Zr_0.7_Ti_0.3_O_3 _composition. EDX analysis of the light-gray secondary phase was not performed since its volume was too small for the analysis to be reliable. However, in a dark-gray area, the phase was found to be ZrO_2_. It was expected that evaporation of Na and Bi might occur which often resulted in a formation of a second phase and compositional inhomogeneity. Similarly, several investigations also found the mentioned loss leading to small existence of the second phase [9,10]. Nevertheless, the amount of the second phase was very low when compared with the matrix phase and therefore could not be detected by the XRD technique.

**Figure 3:**
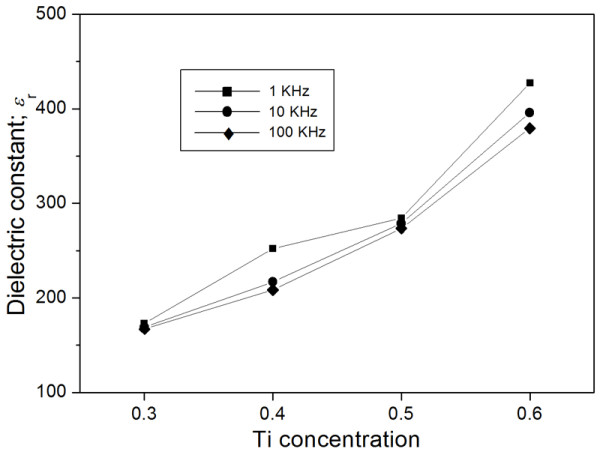
**Dielectric constant at room temperature of BNZT ceramics**. The samples sintered at 950°C were measured at frequencies of 1, 10, and 100 kHz.

Figure 3 displays the compositional dependence of BNZT ceramics of dielectric constant at frequencies of 1, 10, and 100 kHz. All samples showed a decreasing trend of the relative permittivity when the frequency increased. This variation was attributed to the ability of dipoles in following the external field. As the frequency increased, dipoles began to lag behind the field and the value slightly decreased. For BNZT with a varying composition, the values apparently increased with an increment of Ti concentration. Since, in general, polarizability of atoms in a rhombohedral structure was easier than in an orthorhombic lattice, resulting in higher dielectric constant [11], the phase transition of an orthorhombic to a rhombohedral lattice above 0.4 of Ti^4+ ^shown in this study was expected to be the main factor affecting the enhancement of permittivity. In addition, such behavior on dielectric properties at room temperature was similar to that reported by Jaffe et al. [5] and Fujji et al. [12]. For the observed increase in dielectric constant of the BNZT composition containing more than 0.4 Ti content, the decrease of average grain size was believed to partly enhance permittivity values of the samples. In general, a ceramic with smaller grains had higher relative permittivity compared to that with larger grains due to domain wall interactions. The mentioned microstructural feature with improved dielectric constant was also found in several researches [13,14]. Table 2 also listed the dielectric constant of the BNZT ceramic in this work and the BNZ ceramic measured by Lily et al. [1] at frequencies of 1, 10, and 100 kHz. All solid solution compositions exhibited higher dielectric constant values than those of pure BNZ. The improvement suggested that the differences in the crystal structure, i.e., orthorhombic and rhombohedral lattices, as well as ionic size affected directly the increased permittivity of the BNZT ceramic.

**Table 2:** Dielectric constant and loss of the BNZT and BNZ ceramics

*x*	*ε*_r_^a^	tan*δ*^a^ (%)	*ε*_r_^b^	tan*δ*^b^ (%)	*ε*_r_^c^	tan*δ*^c^ (%)	Reference
0	100	-	60	-	25	-	Lily et al.
0.3	173	2.87	169	1.27	167	0.93	This work
0.4	252	5.54	217	2.76	208	2.27	This work
0.5	284	7.98	279	4.32	274	2.56	This work
0.6	427	9.58	396	5.06	379	3.25	This work

*x*, amount of Ti^4+^; *ε*_r_, dielectric constant; tan*δ*, dielectric loss.

**Figure 4:**
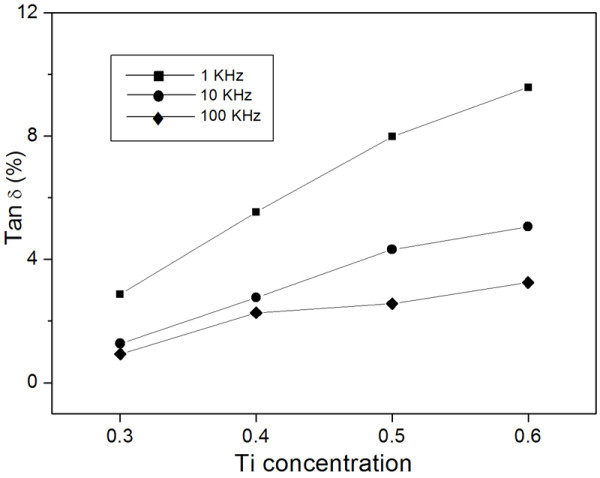
**Dielectric loss at room temperature of BNZT ceramics**. The samples sintered at 950°C were measured at frequencies of 1, 10, and 100 kHz.

Variation of the dissipation factor with various compositions of BNZT materials at different frequencies is presented in Figure 4. It could be noticed that the value decreased while the applied frequency increased. Basically, below 100 kHz, the dielectric loss was progressively higher with the decrease in frequency mainly due to the space-charge polarization phenomena. For the BNZT ceramic with different Zr/Ti ratios, the behavior of dielectric loss showed a similar trend to the dielectric constant, i.e., it increased with increasing addition of Ti. This was the nature of materials having high permittivity that also possessed higher dielectric loss. This study therefore showed that compositional variation in these new BNZT solid solutions affected the crystal structure, phase transition, microstructure, and dielectric properties.

## Conclusions

In this research, BNZT ceramics with Zr/Ti ratios of 0.7:0.3, 0.6:0.4, 0.5:0.5, and 0.4:0.6 were successfully fabricated using a conventional solid-state sintering method. XRD analysis revealed a complete solubility of Ti^4+ ^ions into the B-site of Zr^4+ ^ions for all compositions investigated. Consequently, smaller ions of Ti^4+ ^replacing the host site of Zr^4+ ^ions caused the typical cell volume of BNZ to decrease and produced transformation of an orthorhombic to a rhombohedral lattice above Zr/Ti ratios of 0.6:0.4. As a result, the dielectric constant was enhanced with increasing Ti concentration. Besides, among the BNZT samples possessing a rhombohedral structure, a decrease of average grain size also partly contributed to an increase in the relative permittivity value. In the case of the dissipation factor, the result showed a similar trend to that of the dielectric constant.

## Competing interests

The authors declare that they have no competing interests.

## Authors' contributions

PJ carried out the BNZT ceramic experiments, analysis, and writing of the manuscript. AR carried out the crystal structure investigation of the specimens. SJ and AW participated in the conception and design of the study and revised the manuscript for important intellectual content. All authors read and approved the final version of the manuscript.
